# Rotavirus Strain Trends in United States, 2009–2016: Results from the National Rotavirus Strain Surveillance System (NRSSS)

**DOI:** 10.3390/v14081775

**Published:** 2022-08-15

**Authors:** Slavica Mijatovic-Rustempasic, Jose Jaimes, Charity Perkins, M. Leanne Ward, Mathew D. Esona, Rashi Gautam, Jamie Lewis, Michele Sturgeon, Junaid Panjwani, Gail A. Bloom, Steve Miller, Erik Reisdorf, Ann Marie Riley, Morgan A. Pence, James Dunn, Rangaraj Selvarangan, Robert C. Jerris, Dona DeGroat, Umesh D. Parashar, Margaret M. Cortese, Michael D. Bowen

**Affiliations:** 1 Viral Gastroenteritis Branch, Division of Viral Diseases, National Center for Immunization and Respiratory Diseases, Centers for Disease Control and Prevention, 1600 Clifton Road NE, Mail Stop G-04, Atlanta, GA 30329, USA; 2 Indiana University Health Pathology Laboratory, Indiana University, 350 West 11th Street, Indianapolis, IN 46202, USA; 3 UCSF Clinical Microbiology Laboratory, 185 Berry St, Suite 290, San Francisco, CA 94107, USA; 4 Wisconsin State Laboratory of Hygiene, 2601 Agriculture Drive, Madison, WI 53718, USA; 5 Infectious Disease Diagnostic Laboratory, Boston Children’s Hospital, 300 Longwood Ave., Boston, MA 02115, USA; 6 Cook Children’s Medical Center, 801 Seventh Ave., Fort Worth, TX 76104, USA; 7 Medical Microbiology and Virology, Department of Pathology, Texas Children’s Hospital, 6621 Fannin Street, Suite AB1195, Houston, TX 77030, USA; 8 Children’s Mercy Kansas City, 2401 Gillham Road, Kansas City, MO 64108, USA; 9 Children’s Healthcare of Atlanta, 1405 Clifton Rd, Atlanta, GA 30329, USA; 10 Seattle Children’s Hospital, 5801 Sand Point Way NE, Seattle, WA 98105, USA

**Keywords:** rotavirus, RVA, genotype, prevalence, surveillance, vaccine

## Abstract

Before the introduction of vaccines, group A rotaviruses (RVA) were the leading cause of acute gastroenteritis in children worldwide. The National Rotavirus Strain Surveillance System (NRSSS) was established in 1996 by the Centers for Disease Control and Prevention (CDC) to perform passive RVA surveillance in the USA. We report the distribution of RVA genotypes collected through NRSSS during the 2009–2016 RVA seasons and retrospectively examine the genotypes detected through the NRSSS since 1996. During the 2009–2016 RVA seasons, 2134 RVA-positive fecal specimens were sent to the CDC for analysis of the VP7 and VP4 genes by RT-PCR genotyping assays and sequencing. During 2009–2011, RVA genotype G3P[8] dominated, while G12P[8] was the dominant genotype during 2012–2016. Vaccine strains were detected in 1.7% of specimens and uncommon/unusual strains, including equine-like G3P[8] strains, were found in 1.9%. Phylogenetic analyses showed limited VP7 and VP4 sequence variation within the common genotypes with 1–3 alleles/lineages identified per genotype. A review of 20 years of NRSSS surveillance showed two changes in genotype dominance, from G1P[8] to G3P[8] and then G3P[8] to G12P[8]. A better understanding of the long-term effects of vaccine use on epidemiological and evolutionary dynamics of circulating RVA strains requires continued surveillance.

## 1. Introduction

Prior to the introduction of rotavirus vaccines, group A rotaviruses (RVA) were the leading cause of severe acute gastroenteritis (AGE) in children < 5 years of age worldwide [1]. RVA caused 2.7 million gastroenteritis episodes, 410,000 physician visits, ~250,000 emergency department (ED) visits, 55,000–70,000 hospitalizations, and 20–60 deaths annually, which amounted to approximately USD 1 billion in direct and indirect health care costs in the USA [2]. The American Academy of Pediatrics and Advisory Committee for Immunization Practices (ACIP) recommended two live-attenuated vaccines, RotaTeq^®^ (Merck and Company Inc., Kenilworth, NJ, USA) and Rotarix^®^ (GlaxoSmithKline Biologicals, Rixensart, Belgium), for the routine immunization of all USA children in 2006 and 2008, respectively [3]. In 2009, the World Health Organization recommended both vaccines for the vaccination of all children worldwide. In addition to these two widely used vaccines, other RVA vaccines are available for use on a smaller scale or are being evaluated [4,5,6,7,8,9,10].

RotaTeq^®^ is a pentavalent bovine–human reassortment vaccine that expresses four human RVA VP7 surface antigens (G-genotypes G1, G2, G3 and G4) along with the bovine RVA VP7 G6 antigen as well as human (P[8]) and bovine VP4 antigens (P[5]) [11]. Rotarix^®^ is a monovalent vaccine composed of a single human-derived RVA (strain 89–12), with G1P[8] specificity [12]. Both vaccines are highly effective against RVA-associated hospitalizations and emergency department visits, with protection rates above 80% in the United States [13]. Rates of severe AGE and RVA-related AGE in the USA pediatric population have declined dramatically in the post-vaccine era [14,15,16,17]. The direct and indirect protective effects of vaccination with the RVA vaccines are well documented [18,19,20].

The RVA genome consists of 11 segments of double-stranded RNA (dsRNA), encoding for 6 viral proteins (VP1–4, VP6 and VP7) and depending on the strain, 5 or 6 nonstructural (NSP1-NSP5/6) proteins [21]. Two genes encoding the two outer capsid proteins, VP7 (glycoprotein, G-genotype) and VP4 (protease-cleaved, P-genotype), are typically studied for vaccine effectiveness and epidemiological studies. Historically, a binomial genotyping classification system GxP[x] has been used based on VP7 and VP4 sequence diversity, where x represents the number of the genotype [22]. Although the reassortment of genes could lead to numerous G-P-genotype combinations, only six strains have been associated with the majority of human infections: G1P[8], G2P[4], G3P[8], G4P[8], G9P[8] and G12 in combination with the P[8] or P[6] genotype [23]. The advancement in sequencing technologies allowed for binomial classification to be extended to include all 11 genes, expressed as Gx-P[x]-Ix-Rx-Cx-Mx-Ax-Nx-Tx-Ex-Hx, with x indicating the numbers of the corresponding genotypes [24]. Full-genome sequencing by next-generation sequencing is gradually becoming the standard for genetic classification of RVA; however, most laboratories worldwide still focus on VP7 and VP4 genotyping. Recently, Esona et al. [25] redefined subgenotypic allele constellations for all 11 genes in order to enhance our understanding of RVA evolution. Using the derived within- and between-cluster distances, subgenotype alleles were assigned based on phylogenetic clusters on maximum likelihood trees with bootstrap support of ≥70% and nucleotide identity of ≥95.8% within a cluster.

The National Rotavirus Strain Surveillance System (NRSSS) was established in 1996 by the Centers for Disease Control and Prevention (CDC) to document genotypes circulating in the United States before the introduction of the Rotashield^®^ vaccine (Wyeth Laboratories, Madison, NJ, USA), and continues today [26,27]. A subset of laboratories participating in the National Respiratory and Enterovirus Surveillance System were recruited for passive surveillance through NRSSS to provide aliquots of stool that tested positive for rotavirus antigen by an enzyme immunoassay (EIA) performed as part of routine clinical testing at clinical laboratories across the country. From its onset, the NRSSS-based molecular characterization of RVA genotypes on the two outer capsid protein genes (VP7 and VP4) only using multiplex reverse transcription–polymerase chain reaction genotyping and nucleotide sequencing. Here, we present the binomial genotyping of samples collected through NRSSS that were circulating in the USA between 2009 and 2016 and retrospectively examine RVA genotype trends in the USA as recorded through the NRSSS for the past 20 years. This data set represents the last NRSSS data based solely on EIA and molecular characterization of the VP7 and VP4 genes only. Beginning with the 2017 RVA season, the CDC began performing PCR-based multipathogen testing and full-genome characterization by next-generation sequencing.

## 2. Materials and Methods

The project was conducted over 8 RVA seasons October–December of one calendar year to June–September of the following calendar year, or the fall until the summer (collection dates were not available for all samples): 1 December 2008 through 14 July 2009 (hereafter referred to as 2009); 20 November 2009 through 11 June 2010 (hereafter, 2010); 11 October 2010 through 26 August 2011 (hereafter, 2011); 3 October 2011 through 21 August 2012 (hereafter, 2012); 4 November 2012 through 23 July 2013 (hereafter, 2013); 10 December 2013 through 25 September 2014 (hereafter, 2014); 2 October 2014 through 16 September 2015 (hereafter, 2015); and 21 October 2015 through 30 September 2016 (hereafter, 2016). During the 2009–2016 RVA seasons, 2134 fecal specimens that tested positive by commercially available EIA or lateral flow devices at NRSSS laboratories were sent to the CDC for genotyping. Samples were collected at 12 hospital-based laboratories or state health departments located in Atlanta (GA), Boston (MA), Fort Worth (TX), Indianapolis (IN), Kansas City (MO), Long Beach (CA), Madison (WI), Minneapolis (MN), Omaha (NE), Salt Lake City (UT), San Francisco (CA), and Seattle (WA), as well as a national commercial diagnostic testing laboratory who requested anonymity.

Ten percent stool suspensions were prepared using phosphate-buffered saline (PBS) followed by centrifugation at 3000 × *g* for 10 min. RVA RNA or total nucleic acid (TNA) was extracted from suspension supernatants using two automated extraction systems, the KingFisher™ Flex Purification System (Thermo Fisher Scientific, Waltham, MA, USA) with the MagMAX™-96 Viral RNA Isolation Kit (Thermo Fisher Scientific, Waltham, MA, USA) or MagMAX™ Total Nucleic Acid Isolation Kit (Thermo Fisher Scientific, Waltham, MA, USA), or the MagNA Pure Compact Instrument (Roche Applied Science, Penzberg, Germany) with the MagNA Pure Compact Nucleic Acid Isolation Kit I. Extractions were performed following the manufacturer’s instructions for each kit.

Reverse transcription–polymerase chain reaction (RT-PCR)-based genotyping and sequencing of VP7 and VP4 genes were performed as described previously [27]. In short, the following VP7 and VP4 genotyping primers were used: for VP7, forward primer 9Con1-L in combination with reverse primers 9T1–1, 9T1-Dg, 9T2, 9T3P, 9T-4, and 9T-9B; for VP4, forward primer con3 in combination with reverse primers 1T-1, 1T1-V, 1T1-A, 2T-1, 3T-1, and 4T-1. Six new changes were introduced during this surveillance period: (1) during the 2013 RVA season, a new G and P genotyping system was adopted [20]; (2) analysis of the PCR products was performed on a LabChip^®^ GX instrument (PerkinElmer, Inc., Waltham, MA, USA) in addition to gel electrophoresis as previously described [28]; (3) amplicons generated for sequencing were analyzed and purified on E-gel^®^ SizeSelect cartridges (Thermo Fisher Scientific, Waltham, MA, USA) in addition to gel electrophoresis and column purification, as previously described [27]; (4) purification of the cycle-sequenced product was performed with a magnetic bead protocol [29], or less frequently with the commercially available BigDye XTerminator™ Purification Kit (Thermo Fisher Scientific, Waltham, MA, USA) following the manufacturer’s protocol; (5) samples that could not be amplified by RT-PCR were tested by a real-time RT-PCR (qRT-PCR) assay targeting the NSP3 gene [30]; and (6) samples that tested positive by NSP3 qRT-PCR but did not produce an amplicon with other RT-PCR methods were further amplified using a nested-PCR approach and sequenced [31].

**Figure 1:**
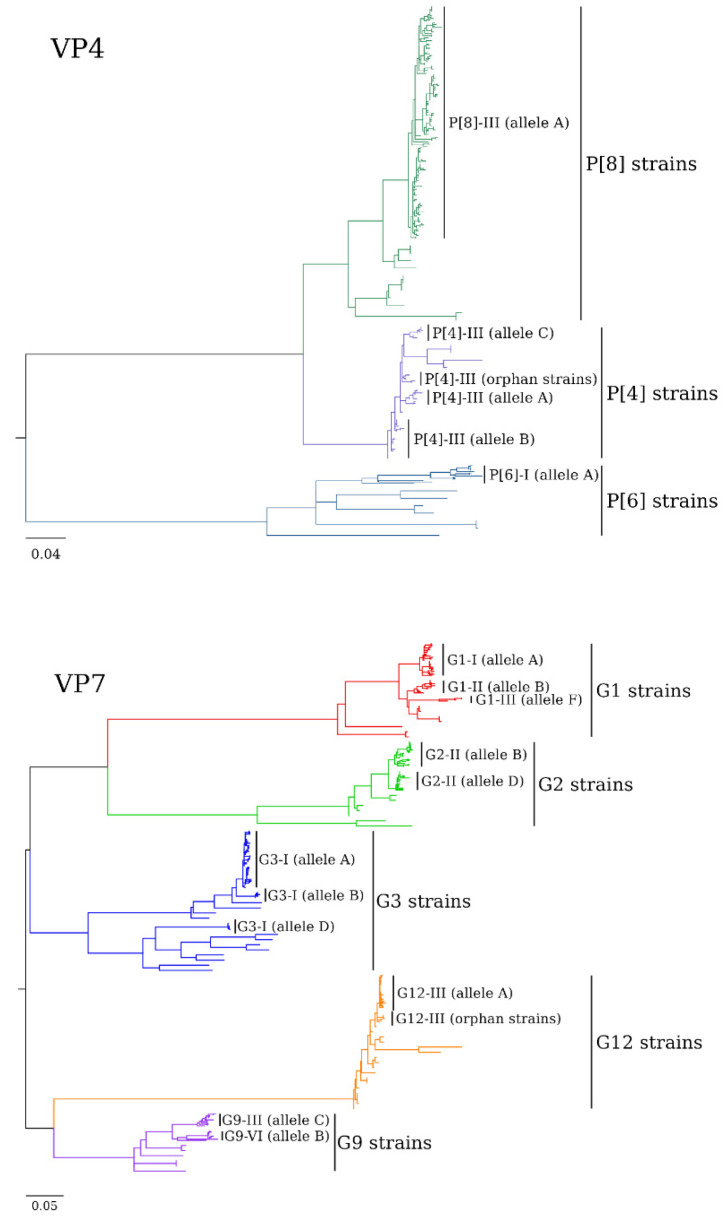
Phylogenetic trees based on aligned nucleotide sequences of the VP7 (585 nt) and VP4 (537 nt) genes of RVAs. Sequence alignments were generated for each genotype using MUSCLE. Top BLAST hits along with lineage reference sequences were also included in these alignments. Optimal models were selected using jModeltest2 using the corrected Aikaike information criterion (AICc). Maximum likelihood trees were generated using PhyML using the approximate likelihood-ratio test (aLRT) option for branch support (see Supplementary Material for aLRT values). Genotype-specific sub lineages are color-coded; genotypes, lineages, and alleles are indicated. Scale bars indicate the number of nucleotide substitutions per site.

Genotypes were assigned based on size of the amplicons as determined by LabChip or gel electrophoretic analysis, or by comparison of sequences obtained in the project with RVA sequences using BLAST v.2.6.0 [32] against a customized database generated using the Virus Variation Resource [33]. Sequences were generated over eight years (2008–2016). All sequences were VP7 and VP4 partial sequences. The final set of sequences used in this report was selected using the following criteria: first, sequences with a minimum length of 600 nucleotides; second, no ambiguous base calls; and last, no frameshifts in the ORF sequence. VP7 and VP4 sequences were identified and annotated using VIGOR [34]. Alignments were created for each gene (VP7 and VP4, Figure 1 and Supplementary Material, Figure S1a,b) using MUSCLE v.3.8 [35]. Top BLAST hits along with lineage reference sequences were also included in these alignments. Maximum likelihood trees were generated in PhyML [34,35] using the approximate likelihood-ratio test (aLRT) option for branch support. Optimal substitution models were selected using the corrected Aikaike information criterion (AICc). Reliability of tree topology was evaluated using aLRT values [36]. Tree diagrams were generated using the GGTREE R package [37]. The VP7 and VP4 sequences were deposited in GenBank under the accession number range MZ545747-MZ546123. Figure 1 shows backbone phylogenetic trees, while the Supplementary Material contains the same VP7 (Figure S1a) and VP4 (Figure S1b) phylogenetic trees at higher resolution with strain labels and aLRT support values.

RT-PCR genotyping was confirmed by sequencing a minimum of 15% of the most prevalent genotype per RVA season (example, G3P[8] during 2009–2011 and G12P[8] during 2013–2016 RVA seasons) and a majority of the less common and rare genotypes. In addition, samples with genotypes that are found in the two RVA vaccines were tested by vaccine detection qRT-PCR, as described previously [38]. Unusual strains initially sequenced by Sanger sequencing for VP7 and VP4 were retrospectively sequenced for all 11 genes (manuscripts in preparation) and will be described in detail later.

All samples that tested negative by all molecular methods were retested with Premier^®^ Rotaclone^®^ (Meridian BioScience, Inc., Cincinnati, OH, USA) following the manufacturer’s instructions to rule out false-positive results from initial antigen detection testing at NRSSS sites.

## 3. Results

Over the eight seasons (2009–2016), 2134 fecal samples that tested positive for RVA at NRSSS sites and the commercial diagnostic laboratory were sent to the CDC for genotyping. Out of the 2134 fecal specimens, 322 (15.1%) tested RVA-negative at the CDC by both Rotaclone and molecular assays (RT-PCR and/or qRT-PCR) and were excluded from further analysis. Table 1 shows a breakdown of the samples by RVA season and city/submitter that tested positive at the CDC (*n* = 1812).

Although this passive system does not attempt to assess the overall national rotavirus disease burden, a biannual pattern, with a lower number of RVA-positive samples submitted during even-numbered years (2010, 2012, 2014, and 2016; number of samples ranged from 37 to 112) and a higher number of positive samples during odd-numbered years (2009, 2011, 2013, and 2015; number of samples ranged from 344 to 417), was observed in this evaluation as has been observed for other USA surveillance systems [39]. The distribution of G and P types of RVA detected in this surveillance period are summarized in Table 2.

In total, both G and P types could be identified for 1776 (98.0%) samples, comprising 29 different wild-type genotype combinations. The remainders were only partially typeable, including 14 (0.8%) strains for which the P type was determined, and the G type remained untypeable and 10 (0.6%) strains for which only the G type could be determined. Both the G and P type were not determined for 12 (0.7%) samples; these samples tested positive for RVA only by qRT-PCR and were EIA negative.

Overall, 1703 (94.0%) of the 1812 strains belonged to one of the six globally important common human genotype combinations (G1P[8], G2P[4], G3P[8], G4P[8], G9P[8] and G12P[8]). With all sites combined, the predominant genotype during the 2009–2011 seasons was G3P[8], representing 27.5% of all positive specimens (range of 1.1–59.9% per season). Genotype G12P[8] replaced G3P[8] as the dominant genotype in 2012 and remained predominant through the 2016 season, representing 44.5% of all positive specimens, with the annual proportion of detection ranging from 0 to 88.2%. The third most frequent genotype was G2P[4] at 12.6%, which ranged from 1.2 to 37.6% per season. Strains G1P[8], G9P[8], and G4P[8] were present at 6.9%, 3.4% and 0.3% overall, respectively. At the site level (Supplementary Material, Figure S2a,b), most sites demonstrated the change from G3P[8] being commonly detected until 2012/2013 when G12P[8] became predominant. Examining odd years (because of the larger number of samples), G1P[8] was the most frequent genotype only in Boston (2009 and 2011), Seattle (2009) and Atlanta (2011). G2P[4] was the most prevalent only in 2011, at three sites (Indianapolis, WI, USA and Long Beach, CA, USA).

Thirty samples (1.7%) were identified as vaccine strains, 28 (1.6%) of which were RotaTeq^®^ and 2 (0.1%) were Rotarix®. Less common genotypes (G1P[4], G2P[6], G2P[8], G3P[6], G3P[4], G3P[9], G4P[6], G6P[8], G6P [14], G8P[4], G8P[14], G9P[4], G12P[6] and G12P[9]) were found in a small percentage (1.9%) of samples (Table 2). A total of nine (0.5%) mixed RVA infections were detected. One mixed infection was a single G-type with two P-types (G2P[4,8]) while other mixed infections could be classified as multiple G-types with a single P-type (G1,3P[8], G1,9P[8], G2,3P[8], G3,9P[8], G4,12P[8], G9,12P[8] and G3,12P[8]).

Sequence comparison and phylogenetic analyses of the VP7 and VP4 genes revealed genotype-specific clustering patterns for strains collected during 2009–2016 season (Figure 1, Supplementary Material, Figure S1a,b).

G1P[8] strains occupied three different VP7 G1 alleles as defined recently by Esona and colleagues [25], with most of the strains in allele A (corresponding to previously identified lineage G1-I) along with common G1 strains circulating in humans presently [40,41]. Two of the 28 RotaTeq^®^ G1 strains sequenced in this project clustered in allele F (G1-III lineage) alongside the RotaTeq-WI79-9/1992/G1P7[5] G1 strain (Figure 1, Supplementary Material Figure S1a). Three G1 wild-type strains from 2010 to 2015 occupied a para-phyletic group that clustered in allele B (lineage G1-II) with the Rotarix^®^ G1 gene. The partial VP7 gene sequences for all wild-type G1 strains have nucleotide and amino acid (669 bp and 223 aa) identities ranging from 88.1 to 100.0% and 90.8–100.0%, respectively.

Phylogenetic analysis showed that all the G2P[4] strains fell into two VP7 alleles within the G2-II lineage [42] (Figure 1, Supplementary Material Figure S1a); the majority of strains collected before 2013 clustered in the larger allele B with other common human strains, while more recent G2 strains, collected after 2013, clustered predominantly with Asian G2 strains in allele D [42,43]. The majority of the G2 strains clustered closest to other G2 strains detected within the same season, and all were closely related with nucleotide similarities (642 bp) ranging from 94.2 to 100.0% and amino acid (214 aa) similarities ranging from 96.2 to 100.0%.

Similarly, phylogenetic analysis determined that all G3 strains, except for two equine-like G3 strains [44], clustered in alleles A and B within the commonly circulating lineage G3-I strains [45], regardless of year of detection (Figure 1, Supplementary Material Figure S1a). Five G3P[6] strains clustered together with other homologous strains in allele B [25], distant from G3P[8] strains in allele A, in an ancestral position relative to other G3 strains (Figure 1, Supplementary Material Figure S1a) [46]. All G3 (allele A and B) strains share nucleotide and amino acid (612 bp and 204 aa) similarities ranging from 94.3 to 100.0% and 95.0 to 100.0%, respectively. Two G3P[8] samples detected in 2015 and 2016, respectively, were identified to be equine-like G3P[8] strains similar to those that were detected first in Australia in 2013 [47]. They cluster with the other equine-like G3P[8] strains within the allele D (G3-II lineage) [25]. These two strains were determined by the conventional RT-PCR genotyping assay to be the G3P[8] genotype and identified as equine-like G3P[8] strains by Sanger sequencing of the VP7 and VP4 genes. Subsequently, their full genomes were sequenced (accession numbers MF997040 [44]; MZ546112).

Four G9P[8] strains detected during the 2013 season clustered within the VP7 G9 allele B (lineage G9-VI) [25,48], which consisted exclusively of Asian strains, while three other G9 strains clustered in allele C (lineage G9-III) with common human G9 strains (Figure 1, Supplementary Material Figure S1a) [46,48]. All G9 strains identified in this report shared nucleotide and amino acid (705 bp and 235 aa) similarities ranging from 90.7 to 100.0% and 95.3 to 100.0%, respectively.

The majority of G12 RVA strains from this project clustered in allele A (G12-III lineage) with other G12 strains that have been detected worldwide in human populations [49]. Within lineage G12-III, strains sequenced in this project can be further divided in three sublineages: the older G12-III sublineage contained most of the strains collected through the 2011 season, while most strains collected starting in 2012 season clustered into the two newer G12-III sublineages (Figure 1, Supplementary Material Figure S1a). Four strains described as G12-III lineage strains could not be assigned to any of the described alleles; therefore, we denoted them as G12-III orphan strains. In addition, four strains did not cluster within any of the previously defined lineages. All G12-III strains identified in this project shared nucleotide and amino acid (603 nt and 201 aa) similarities ranging from 94.8 to 100.0% and 95.5 to 100.0%, respectively.

Uncommon strains (G1P[4], G2P[6], G2P[8], G3P[4], G3P[6], G3P[9], G4P[6], G6P[8], G6P[14], G8P[4], G8P[14], G9P[4], G12P[6] and G12P[9]) were not included in the phylogenetic analysis because they were previously described elsewhere or will be described in future publications [50,51,52,53].

The wild-type P[8] strains (G1P[8], G3P[8], G9P[8] and G12P[8]) all clustered into allele A (lineage P[8]-III; Figure 1, Supplementary Material Figure S1b) [54,55,56] regardless of the year of detection, with nucleotide and amino acid (537 bp and 179 aa) similarities ranging from 94.9 to 100.0% and 94.8 to 100.0%, respectively.

Similarly to P[8] strains, all P[4] (G2P[4]) strains and P[6] (G3P[6]) strains clustered within single previously established lineages, P[4]-III (P[4] alleles A, B and C) and P[6]-I (P[6] allele A), respectively, (Figure 1, Supplementary Material Figure S1b) [46,57,58]. Nucleotide and amino acid (579 bp and 183 aa) similarities among P[4] strains ranged from 95.9 to 100.0% and 97.0 to 100.0%, while among P[6] strains, nucleotide and amino acid (651 bp and 217 aa) similarities ranged from 93.6 to 100.0% and 94.8 to 100.0%, respectively. Within the P[4]-III lineage, there are four sublineages, corresponding to previously well-defined alleles A, B and C, while two P[4] strains cluster with strains which were previously not assigned to an allele, and, therefore, were designated as P[4]-III (orphan strains). Allele C strains cluster with P[4] strains from Asia which are associated with the G2 or G9 genotype [59] (Figure 1, Supplementary Material Figure S1b).

**Figure 2:**
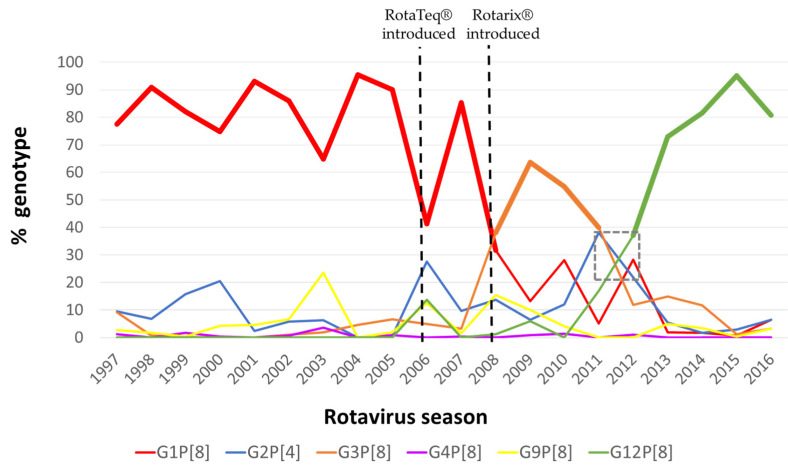
The percent distribution of six major genotypes (G1P[8], G2P[4], G3P[8], G4P[8], G9P[8] and G12P[8]) during the 1997–2016 seasons; data from 1997 through 2008 seasons were obtained from previously published reports. Rarer genotypes were not accounted for in this graphical representation. Percentages were calculated using samples which tested positive by EIA at the collection site and were confirmed at the CDC. Percentage of common genotypes was adjusted to equal 100% per season. Several co-dominant genotypes were detected at comparable levels during the 2011 and 2012 RV seasons, denoted on the graph with a gray dashed box. The licensure year of each RVA vaccine is denoted by the black dashed vertical lines.

A review of NRSSS surveillance data over 20 years revealed the dominating prevalence of the RVA genotype G1P[8] during the 1997–2007 seasons (Figure 2).

Examining the distribution of genotypes with all sites combined, it appears a genotype shift occurred in the 2008 season, when the G1P[8] genotype was replaced by G3P[8] which remained the predominant genotype for four years. Two RVA seasons, 2011 and 2012, were the most diverse due to comparable prevalences of the G1P[8], G2[4], G3P[8] and G12P[8] genotypes. During the 2012 RVA season, however, genotype G12P[8] rose sharply to establish itself as the most abundant genotype. During the season when genotypes G1P[8] and G12P[8] were predominant, both were detected at high percentages of 95.4% and 95.0%, respectively, whereas genotype G3P[8] prevalence never exceeded 65%. Several genotypes were represented at comparable levels during the 2011 and 2012 seasons, when the shift from G3P[8] to G12P[8] predominance occurred. This shift was more gradual compared to the genotype shift from G1P[8] to G3P[8] during the 2007–2008 RVA season, which occurred abruptly and during which other major genotypes stayed at levels comparable to previous seasons.

## 4. Discussion

This evaluation presents NRSSS data from the period December 2008 to September 2016 that describe the distribution of genotypes causing RVA acute gastroenteritis in the USA. We demonstrated that a genotype shift from G3P[8] to G12P[8] occurred during the surveillance period. This genotype shift is consistent with findings from other US surveillance systems [39,60] as well as many countries [61,62,63,64,65] that found an increased incidence of genotype G12 during the 2012 RVA season; more specifically, the G12 lineage-III (allele A) associated with the P[8] genotype, although G12P[6] was found to be present at lower levels [18,19]. Phylogenetic analyses showed limited sequence (VP7 and VP4) variation within the common genotypes; most wild-type strains belonged to one lineage, with the exception of G1, G3 and G9 strains, which clustered within 2–3 lineages. The allele designations [25] provided further insight into the diversity of these genotypes. We found alleles among genotypes G2, G9 and P[4] which contained only our report strains and RVA strains from Asia. Additionally, we detected equine-like G3P[8] strains like these that originated in Australia [47], suggesting that new genotype variants emerging from South East Asia and Australia might be a source of new genotypes observed in the USA now and in the future. Furthermore, assigning alleles helped us differentiate between the G3 strains based on their associated P-genotype, and we found clear clustering patterns between the G3P[8] (allele A) and G3P[6] (allele B) strains. However, we found G12 strains that did not cluster well within previously defined lineages (four G12 strains that did not cluster with strains defined within any of the G12 lineages) or alleles (three G12-III lineage orphan strains). We also found P[4] strains that clustered within a single lineage, but did not cluster with any of the previously defined alleles. This observation of orphan strains either not clustering within the lineages and/or the alleles could most likely be due to the use of the partial sequences for phylogenetic analysis in this report instead of whole-gene sequences used previously for allele assignment. Similar to previously published US Surveillance studies [27,39], unusual genotypes and RVA mixed genotypes were identified in small proportions during this surveillance period.

The number of RVA-positive samples submitted to the CDC varied by RVA season and ranged from 37 to 417 samples submitted (Table 1 and Table 2). A biennial pattern of low RVA incidence during even years (i.e., 2010, 2012) and high RVA incidence during odd years (i.e., 2009, 2011) likely directly influenced the numbers of samples submitted for this project. As stated by Pindyck et al., the shift from annual to biennial RVA peak activity after the introduction of RVA vaccines is attributed to an insufficient number of RVA-infected children to support annual transmission [13]. Although the RVA vaccination coverage has increased (estimated at 73.2% in 2015 [13]), a proportion of children remain unvaccinated, and in low-RVA seasons, these unvaccinated children might not be exposed to wild-type RVA infections and remain susceptible in their second year of life. Together with unvaccinated children from the next birth cohort, they form a larger group of susceptible children to produce increases in RVA infection in alternate years. This phenomenon was predicted previously by modeling of RVA transmission in the post-vaccine licensure era and reflects the nonlinear feedback of herd immunity [66]. Vaccine strain detection levels varied by season as well (0–8.2%), with RotaTeq^®^ being more frequently detected than Rotarix^®^. The highest levels of vaccine strain detection was observed in “even” years when RVA disease activity was lower and fewer samples were submitted to the CDC.

Examining all sites combined, we observed two temporal changes in genotype dominance: a shift from G3P[8] to G12P[8] in 2012 and an earlier shift from G1P[8] to G3P[8] during the 2008 season after introduction of RotaTeq^®^. Temporal and geographical differences between the RVA genotypes have been extensively documented [67,68,69]. The predominant genotype G1P[8], which circulated along with four other genotypes (G2P[4], G3P[8], G4P[8] and G9P[8]) during the 1980s to early 2000s, accounted for approximately 75% of all strains worldwide [68,70]. A global decline in G1P[8] strains coincided with the emergence of G3P[8] strains followed by the emergence of G12P[8] strains in many regions in recent years [67,71,72]. Similarly, USA genotyping data obtained through an active surveillance system, the New Vaccine Surveillance Network, showed a dramatic genotype change from G1P[8] to G3P[8] in 2009 [73] and then to G12P[8] in 2012 [39]. Globally, a rise in G12 strains in the post-vaccine era (early 2010) has been reported in countries using both vaccines and those who have yet to introduce vaccination [30,39,59,62,63,64,65,67,68,74,75,76,77,78,79], suggesting that this shift in genotypes is a result of natural genotype cycling rather than a result of vaccine-selective pressures [22,68. Both RotaTeq and Rotarix have demonstrated good effectiveness against G12P[8] disease for the combined ED/hospitalization outcome in the NSVN platform [11,20]. A recent report examining antigenic epitopes of VP7 proteins of G12P[8] strains found that they differed markedly from those of vaccine strains and that fully vaccinated children were infected with G12P[8] strains more frequently than with other RVA genotypes [59]. Similar observations were made by McDonald and colleagues who found that contemporary G12 strains occupied two sublineages within the G12-III lineage and differed at the nucleotide but not the amino acid level, suggesting that G12 strains diverged prior to the estimated time of G12 introduction into the USA in 2000 [80]. This report emphasizes the need for continued monitoring of RVA vaccine efficacy against emerging RVA genotypes.

After the introduction of Rotarix^®^ in Brazil (in 2006) and several Australian states (in 2007), a shift to G2P[4] strains was observed compared to previous RVA seasons [81,82,83,84,85], whereas states in Australia that started using the RotaTeq^®^ vaccine recorded an increase in G3P[8] genotypes [85]. The hypothesis that two distinctive trends (G3P[8] and G2P[4] predominance after RotaTeq^®^ and Rotarix^®^ introduction, respectively) are due to vaccine pressure is challenged by other surveillance findings, such as: the re-emergence of G2P[4] immediately after the introduction of RotaTeq^®^ [86,87]; a high prevalence of the G2P[4] genotype in Latin American countries irrespective of RVA vaccine introduction [87,88,89,90]; and a global rise in G12 strains regardless of vaccine introduction [63,67,68,91]. Recent reports from Australia highlight a distinctive pattern in genotype prevalence between states that use either Rotarix^®^ or RotaTeq^®^ exclusively: continued dominance of G12P[8] strains in RotaTeq^®^ states and codominance of G2P[4] and equine-like G3P[8] in states and territories using Rotarix^®^ [92]. In addition, a more recent report from New South Wales in Australia, where Rotarix^®^ is used exclusively, identified equine-like G3P[8] and G8P[8] to be the most common genotypes in children older than 6 months [93]. A shift from G1P[8] to G3P[8] occurred 2 years after the introduction of RotaTeq^®^ and during the year Rotarix^®^ was introduced in the USA and it occurred in a single season, whereas a shift from the G3P[8] to the G12P[8] genotype occurred years after the introduction of these two vaccines and it occurred over the two RVA seasons. RotaTeq^®^ is more widely used in the USA [16]. Shifts in RVA genotypes are probably the result of both immune pressure from vaccines and natural genotype cycling.

RVA genotype distributions fluctuate both geographically and temporally in the absence of the vaccine. Natural variation among RVA strains with sometimes rapid changes over successive RVA seasons has been observed in many locations [39,67]. Determining whether a change in circulating genotypes, after the introduction of a vaccine, is a result of natural seasonal shift or vaccine pressure is challenging due to these naturally occurring changes in RVA genotypes. It has been postulated that if the vaccine is highly effective against the X genotype but lower against the Y genotype, then the absolute numbers of both genotypes will decrease, but the Y:X ratio will increase among the remaining RVA strains, leading to a shift in genotypes in favor of Y strains [94]. Regardless of whether genotype shifts observed globally are due to natural variation or vaccine pressure, the need for continual monitoring of RVA genotypes remains.

Following oral vaccination with either RVA vaccine, vaccine strains can replicate in the gastrointestinal tract and may be shed in the stool [95,96,97]. Shedding of vaccine strains following RotaTeq^®^ vaccination was reported for up to 6 months in nonsymptomatic, nonimmunocompromised children [98] and ≥200 days in immunocompromised children [99]. Shedding of Rotarix^®^ has been reported for up to 2 months post vaccination [100,101]. Horizontal transmission of Rotarix^®^ or RotaTeq^®^ from vaccinated to unvaccinated persons can occur in the absence of gastrointestinal symptoms, and can result in symptomatic RVA gastroenteritis requiring care [100,102,103,104,105]. In addition to horizontal transmission of vaccine strains, the major concern regarding vaccination with live attenuated vaccines is the potential of the vaccine strains to evolve and cause disease [103,106,107]. A low occurrence of vaccine strains was found in our evaluation and may only represent shedding from infants after vaccination; overall, less than 2% of strains tested positive with qRT-PCR targeting the Rotarix^®^ NSP2 and VP4 genes, as well as RotaTeq^®^ VP6 and VP3 genes. No sample was found to contain vaccine strains (either Rotarix^®^ or RotaTeq ^®^) in combination with a wild-type strain when VP7 and VP4 genes were analyzed. Reassortment or the coinfection of the vaccine with wild-type RVA strains was not evaluated based on all 11 genes.

The CDC established the National Rotavirus Strain Surveillance System (NRSSS) in 1996 in collaboration with laboratories across the United States, as a passive surveillance system to document the genotype circulation before and after introduction of the Rotashield^®^ vaccine. A subset of laboratories participating in the National Respiratory and Enterovirus Surveillance System provides aliquots of stool that tested positive for rotavirus antigen by enzyme immunoassay (EIA) performed as part of routine clinical testing at clinical laboratories across the country [26,27]. Limited patient information is collected to include a sample collection date and/or a patient date of birth, while severity of illness, hospitalization or rotavirus vaccination status are not obtained. The low-cost NRSSS provides insight into long-term rotavirus-genotyping data in the US; however, it does not provide in-depth epidemiological and patient data. In contrast, the high-cost New Vaccine Surveillance Network (NVSN) was established by the CDC in 2006 as an active, prospective, population-based acute gastroenteritis surveillance system, in anticipation of two rotavirus vaccines, RotaTeq^®^ and Rotarix^®^. Using a standardized questionnaire administered to the consenting parents and medical chart reviews, in-depth patient data are collected to include demographic and socioeconomic data, as well as medical history and vaccination status [73]. Both surveillance systems, NRSSS and NVSN, are beneficial in assessing circulating RVA genotypes in the USA and, for genotype tracking, yield similar results.

There are limitations of this report. A first major limitation is that the NRSSS is a passive surveillance system, with only limited clinical information collected for each case. Because it is a passive system providing residual stool samples after rotavirus testing that was ordered at the discretion of a clinician, the numbers of rotavirus-positive samples provided by a site do not necessarily correlate with the burden of rotavirus disease at that location. Secondly, we summarized the prevalence of genotypes from all sites combined, so for any given season, the results are more representative of the sites providing the larger numbers of samples. Thirdly, RVA-negative samples were not examined for other gastrointestinal pathogens and were excluded from the evaluation, and RVA-positive samples were not screened for other pathogens. To address this limitation in future studies, starting in the 2017 RVA season, all NRSSS samples were screened with the multi-pathogen xTAG^®^ Gastrointestinal Pathogen Panel on the MAGPIX^®^ System (Luminex Corporation, Northbrook, IL, USA) to detect other gastroenteritis pathogens in addition to RVA. Fourthly, information on circulating strains was limited to two genes that were partially sequenced and did not provide insight into full genome constellations. Our partial VP7 and VP4 sequence analysis assigned our sequences to lineages and alleles previously described [42,108,109] but our allele designations may not align with alleles established by Esona et al. which were based on full gene sequences [25]. To address this limitation for the future studies, our lab extended sequencing from two genes to all 11 RVA genes starting with the 2017 RVA season. Fifthly, the number of samples per NRSSS site was small for geographical differences to be assessed. Sequencing of the complete RVA genome and monitoring gastrointestinal multipathogen trends will be necessary to better understand RVA epidemiology and biology as well as to improve vaccination strategies against RVA disease. Lastly, an improved RT-PCR genotyping system, which includes the first season of G12P[8] predominance, was used to generate genotyping data presented here starting in 2013. Results generated with the new genotyping system were confirmed by previously used RT-PCR assays as well as nucleotide sequencing, to rule out the possibility that the observed changes in the genotype prevalence were an artifact of a new genotyping system.

## 5. Conclusions

In conclusion, the RVA genotype landscape in the United States has changed over the last 20 years. Although changes in a major RVA genotype do not occur frequently and unusual and mixed genotypes (as well as vaccine strains) are detected at low levels, continuing RVA genotype surveillance is necessary for many reasons. RVA vaccines are live vaccines; vaccine genes can reassort with vaccine and/or wild-type RVA strains to cause illness [102,106]. Elimination of RVA, unlike polio or measles, seems unrealistic in the near future; nonhuman mammals can serve as hosts for RVA, allowing for the reassortment of human and animal genes and the introduction of animal RVA genes into RVA strains infecting humans. Additionally, if RVA surveillance ceases, and a resurgence of RVA disease occurs later, surveillance platforms and systems would require time to resume in order to answer which genotype(s) are causing the disease. Finally, formulation of future RVA vaccines will depend on knowledge of genotypes associated with disease today. Therefore, continuing surveillance studies are needed to monitor the dynamics of RVA genotype circulation in the USA.

## Figures and Tables

**Table 1:** Number of Positive Samples Submitted by RVA Season and City, 2009–2016 (*n* = 1812).

	Season								
City/Submitter	2009	2010	2011	2012	2013	2014	2015	2016	Total
Atlanta, GA	25	-	23	4	30	12	21	-	**115**
Boston, MA	22	6	32	-	-	1	19	1	**80**
Fort Worth, TX	44	5	41	1	45	29	-	6	**171**
Indianapolis, IN	40	8	55	-	45	6	60	-	**215**
Kansas City, MO *	45	13	59	-	56	3	44	-	**221**
Long Beach, CA	91	-	20	7	53	-	18	-	**188**
Wisconsin State Lab, WI	-	-	64	14	54	6	60	6	**204**
Minneapolis, MN	20	-	-	-	-	-	-	-	**20**
Omaha, NE	16	-	30	-	-	-	-	-	**46**
Salt Lake City, UT	-	18	-	-	-	-	-	-	**18**
San Francisco, CA	32	6	19	5	6	-	25	2	**96**
Seattle, WA *	27	24	32	15	34	5	12	8	**155**
Commercial Laboratory	-	-	-	66	94	23	85	14	**283**
**TOTAL**	**362**	**80**	**375**	**112**	**417**	**85**	**344**	**37**	**1812**

* Sites that participate in both the National Rotavirus Strain Surveillance System (NRSSS) and the New Vaccine Surveillance Network (NVSN).

**Table 2:** G and P Genotyping Results of RVA Strains in the United States during 2009–2016.

RVA Strain	Number (%) of Strains by Season								
	2009	2010	2011	2012	2013	2014	2015	2016	Total
**Common**									
G1P[8]	45 (12.4)	21 (26.3)	19 (5.1)	26 (23.2)	8 (1.9)	1 (1.2)	2 (0.6)	2 (5.4)	**124 (6.9)**
G2P[4]	22 (6.1)	9 (11.3)	141 (37.6)	20 (17.9)	22 (5.3)	1 (1.2)	9 (2.6)	2 (5.4)	**226 (12.6)**
G3P[8]	217 (59.9)	41 (51.3)	148 (39.5)	11 (9.8)	61 (14.6)	7 (8.2)	5 (1.1)	1 (2.7)	**491 (27.5)**
G4P[8]	3 (0.8)	1 (1.3)	-	1 (0.9)	-	-	-	-	**5 (0.3)**
G9P[8]	34 (9.4)	3 (3.8)	-	-	20 (4.8)	2 (2.4)	1 (0.3)	1 (2.7)	**61 (3.4)**
G12P[8]	20 (5.5)	-	63 (16.8)	34 (30.4)	298 (71.5)	49 (57.6)	307 (88.2)	25 (67.6)	**796 (44.5)**
**Vaccine**									
Rotateq^®^	10 (2.8)	-	2 (0.5)	5 (4.5)	1 (0.2)	7 (8.2)	3 (0.9)	-	**28 (1.6)**
Rotarix^®^	-	-	-	1 (0.9)	-	-	1 (0.3)	-	**2 (0.1)**
Uncommon *	4 (1.1)	1 (1.3)	2 (0.5)	8 (7.1)	2 (0.5)	6 (7.1)	7 (2.0)	4 (10.8)	**34 (1.9)**
Mixed **	2 (0.6)	4 (5.0)	-	-	3 (0.7)	-	-	-	**9 (0.5)**
Untypeable ***	5 (1.4)	-	-	6 (5.4)	2 (0.5)	12 (14.1)	9 (4.0)	2 (5.4)	**36 (2.0)**
**TOTAL**	**362**	**80**	**375**	**112**	**417**	**85**	**344**	**37**	**1812**

* During the 2009 season, 1 G8P[4] and 3 G2P[6] strains were detected. During 2010, one G12P[6] strain was detected. During 2011 RV, a G3P[6] and G9P[4] strain were detected. During 2012, 2 G3P[6], G2P[6], G3P[4], G3P [9], G6P[8], G8P[14], and G12P[6] were detected. During 2013, 2 G12P [9] were detected. During 2014, 5 G3P[6] and 1 G2P[8] were detected. During 2015, 2 G1P[4], 2 G3P[6], 2 G6P[14], and 1 G4P[6] were detected. During 2016, 2 G9P[4] and 2 G8P[14] were detected. ** During the 2009 season, one G2P[4,8] strain and one G2,3P[8] strain were detected. During 2010, one G1,3P[8], one G1,9P[8], one G3,9P[8], and one G3,12P[8] were detected. During 2013, one G4,12P[8], one G9,12P[8], and one G3,12P[8] were detected. *** During the 2009 season, 3 G1P[nt], 1 GntP[8], and 1 GntP[nt] were detected. During 2012, 6 GntP[6] strains were detected. During 2013, two G3P[nt] strains were detected. During 2014, 5 GntP[nt], 4 G3P[nt], 2 GntP[8], and 1 G12P[nt] were detected. During 2015, 6 GntP[nt], 2 GntP[8], and 1 GntP[4] strains were detected. During 2016, two GntP[8] were detected.

## Data Availability

Not applicable.

## Supplementary Materials

The following supporting information can be downloaded at: https://www.mdpi.com/article/10.3390/v14081775/s1. Figure S1a,b. Phylogenetic trees based on aligned nucleotide sequences of the VP7 (585 nt) and VP4 (537 nt) genes of RVAs, respectively. Sequence alignments were generated for each genotype using MUSCLE. Top BLAST hits along with lineage reference sequences were also included in these alignments. Optimal models were selected using jModeltest2 using the corrected Aikaike information criterion (AICc). Maximum likelihood trees were generated using PhyML using the approximate likelihood-ratio test (aLRT) option for branch support shown as a proportion at nodes. Genotype-specific sub lineages are color-coded and genotypes, lineages, and alleles are indicated. Current report strains are indicated by circles at branch termini. Scale bars indicate the number of nucleotide substitutions per site. Figure S2a,b. Percentage distribution of major Genotyping Results per city/submitter during odd years (2009, 2011, 2013 and 2015) for: (2a) Atlanta, GA, Boston, MA, Indianapolis, IN, Wisconsin, WI, Kansas City, MO, Omaha, NE, (2b) Fort Worth, TX, Seattle, WA, Long Beach, CA, San Francisco, CA and a National Commercial Laboratory.
