# Supplementary figures and images for: Rotavirus Strain Trends in United States, 2009–2016: Results from the National Rotavirus Strain Surveillance System (NRSSS)
Source: Viruses. 2022 Aug 15;14(8):1775. doi: 10.3390/v14081775 (PMC9414880; doi:10.3390/v14081775)

Figure S1a

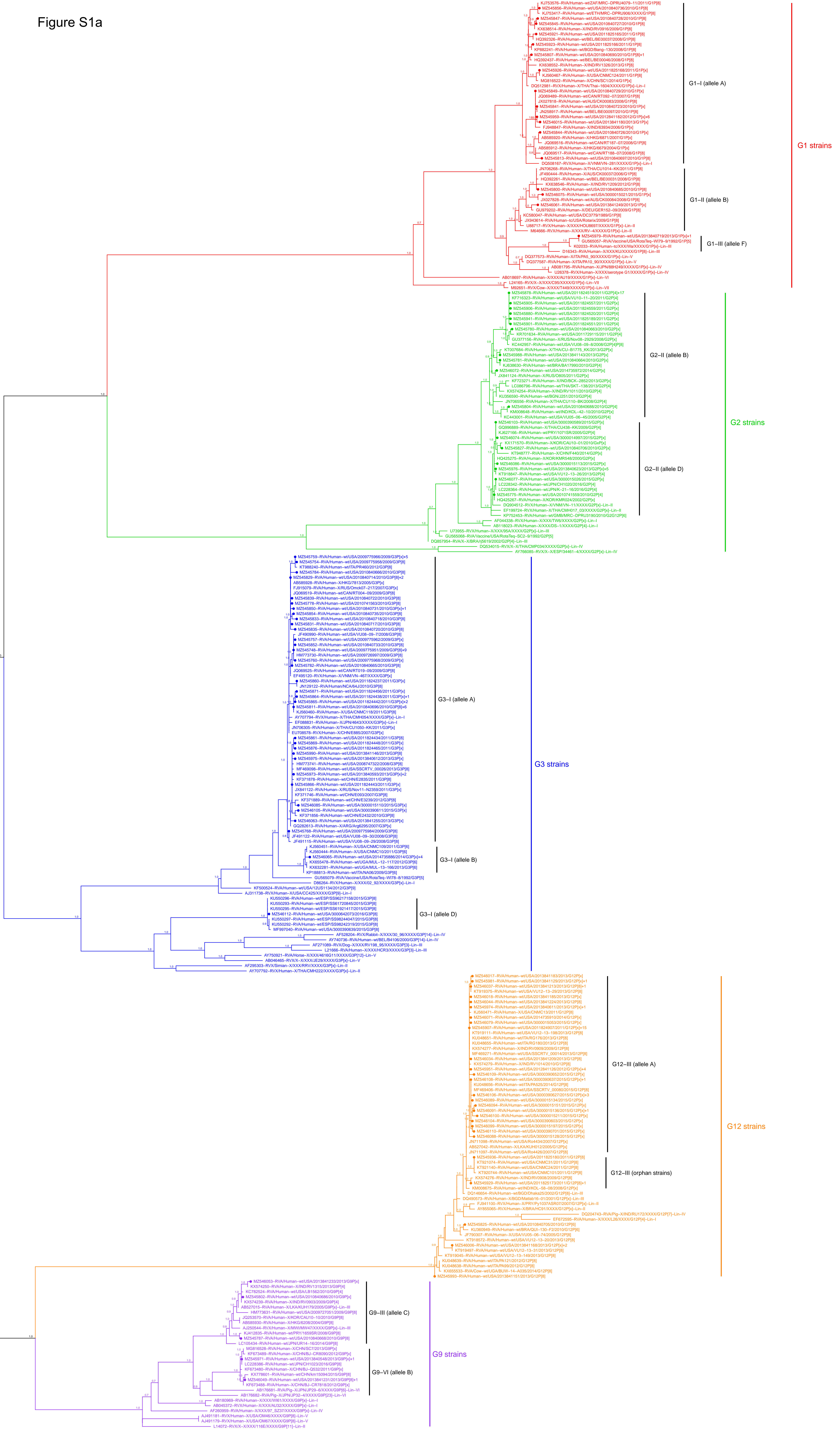

Figure S1b

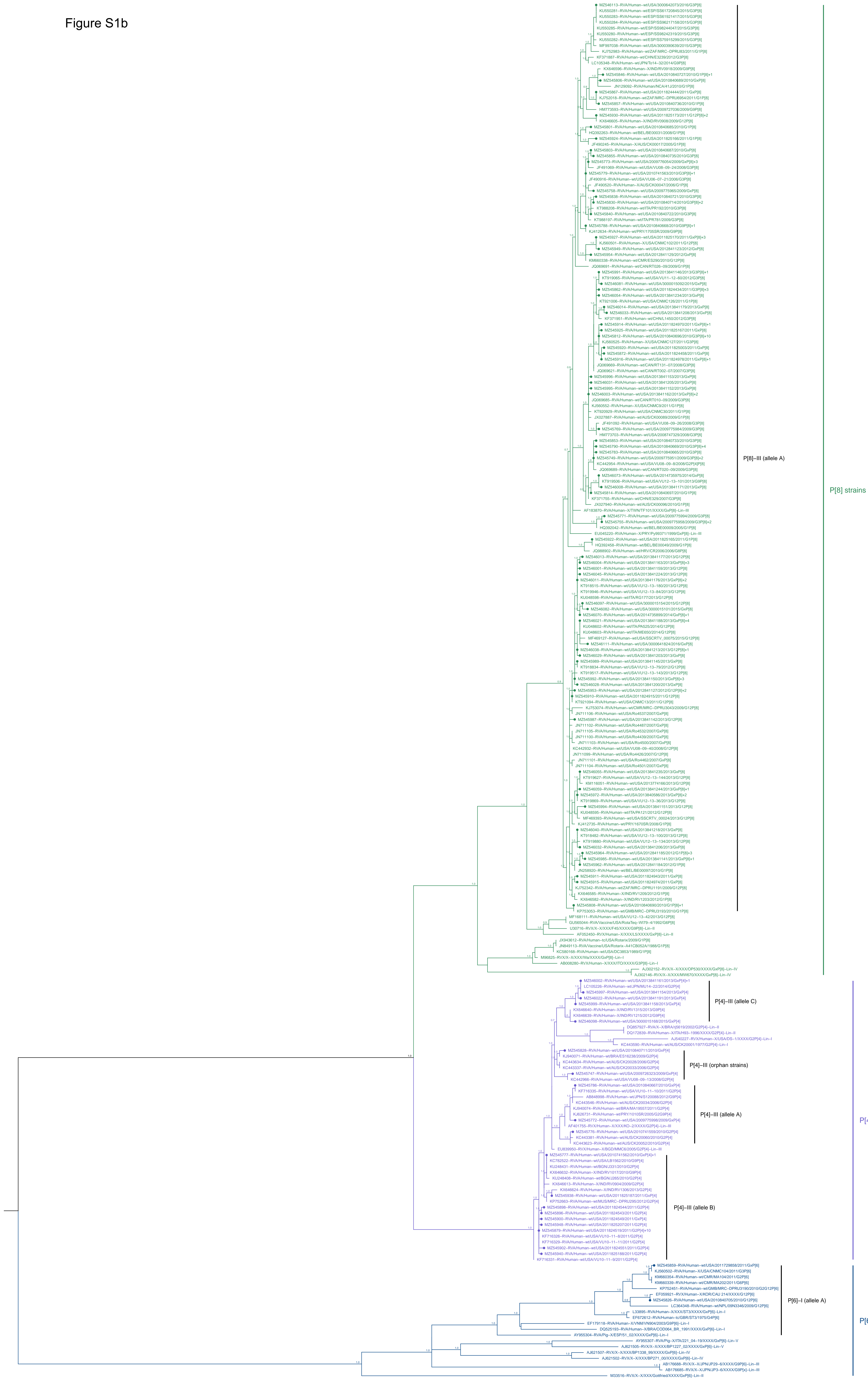

Figure S2a

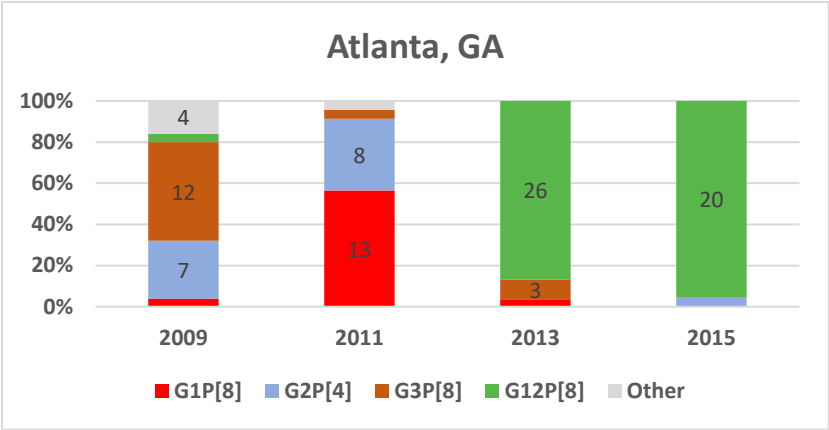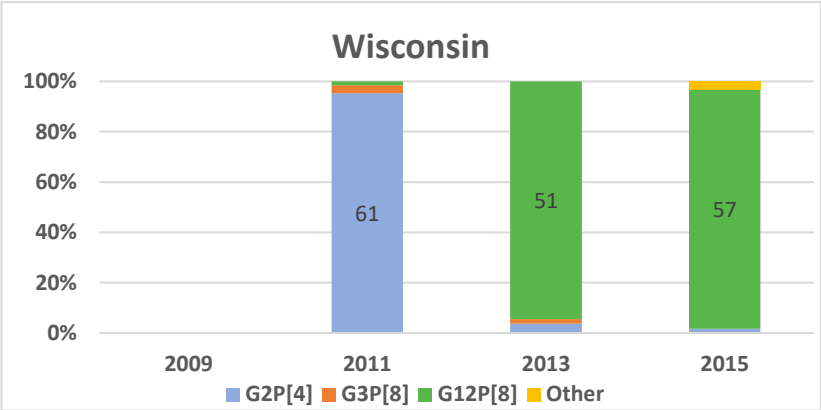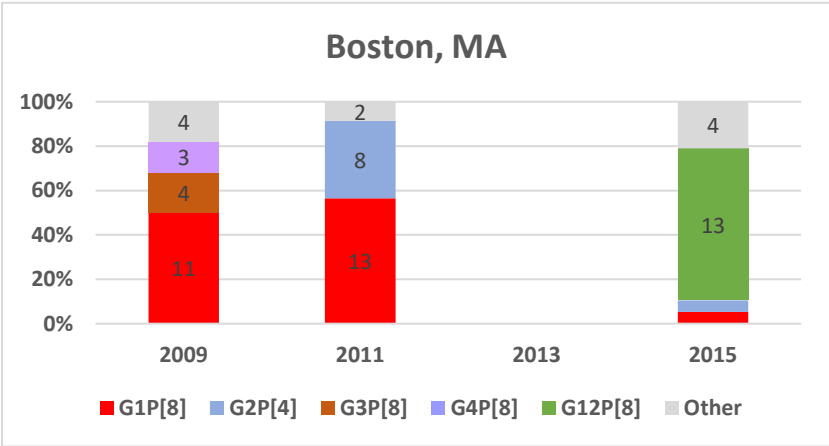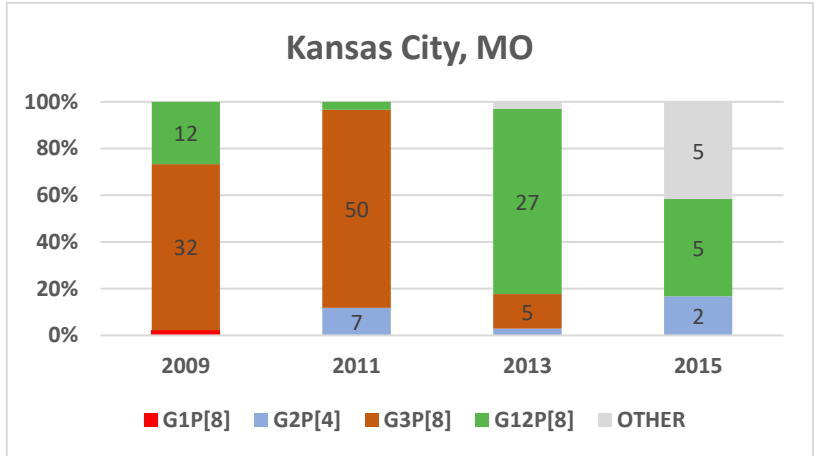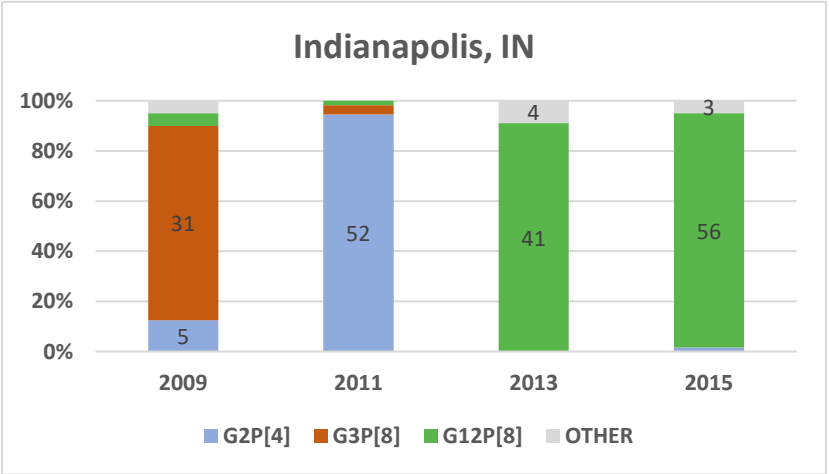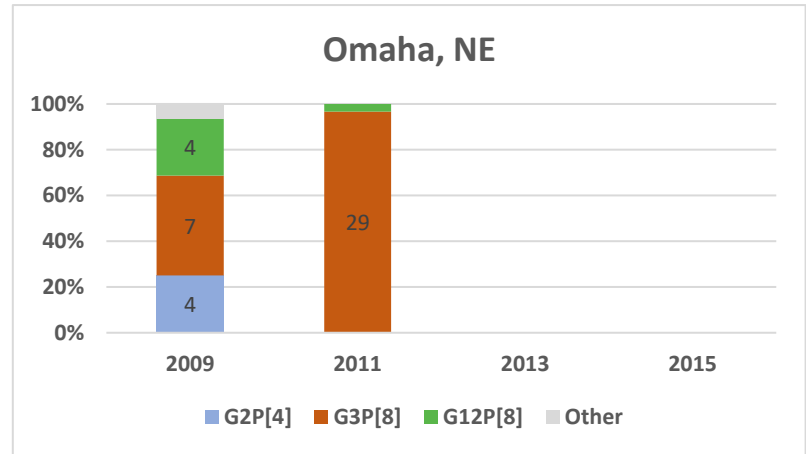

Figure S2b

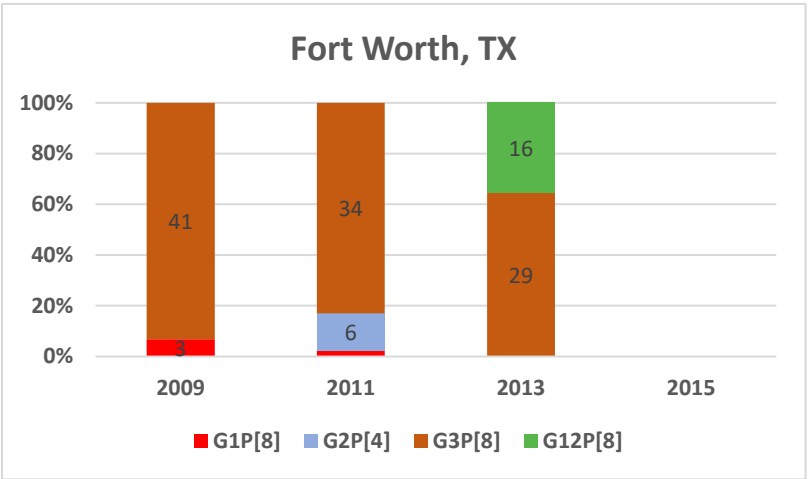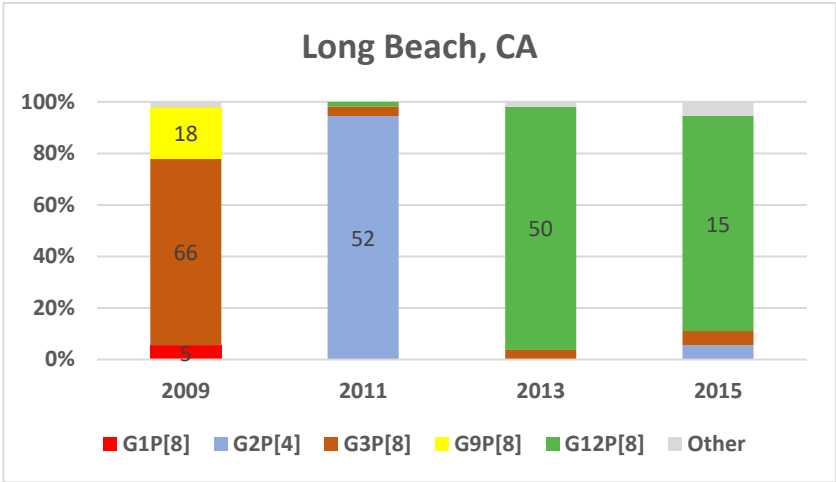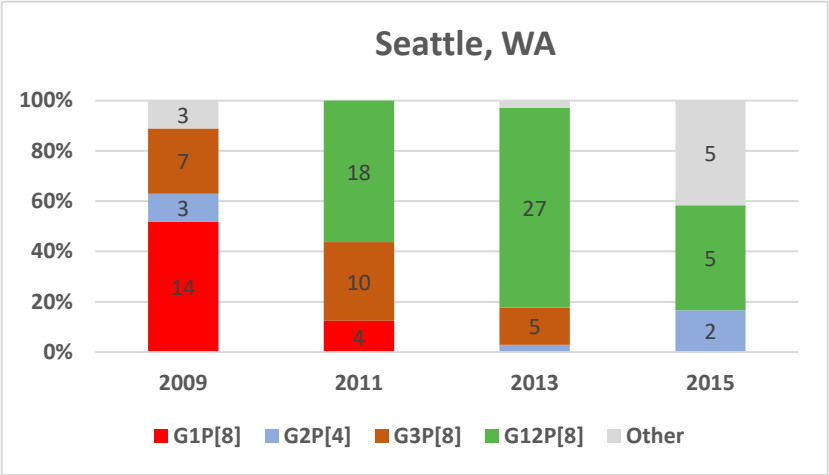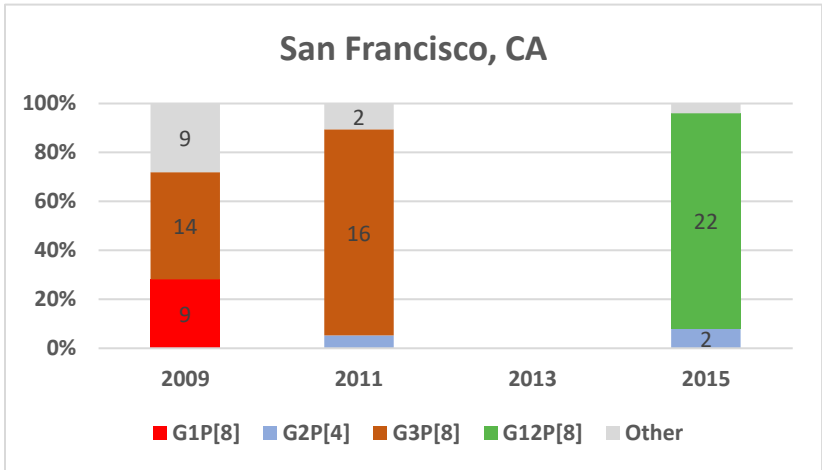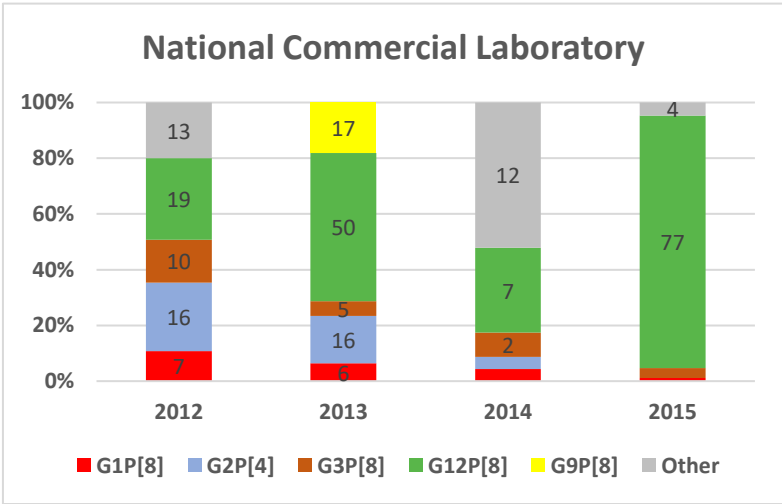

Supplement: Supplementary file 1 [file viruses-14-01775-s001.zip › viruses-1811900-supplementary.pdf]
